# Prosociality predicts changes in leisure activities during the COVID-19 pandemic

**DOI:** 10.3389/fpsyg.2024.1320885

**Published:** 2024-02-27

**Authors:** Naoki Konishi, Motohiro Kimura, Yuji Takeda

**Affiliations:** National Institute of Advanced Industrial Science and Technology, Tsukuba, Japan

**Keywords:** prosociality, personality trait, COVID-19, behavioral changes, leisure activity, well-being

## Abstract

Several studies suggest that leisure activities enhance well-being. In line with this perspective, a recent study indicates that augmenting indoor leisure activities to compensate for diminished outdoor pursuits could sustain or enhance well-being during the COVID-19 pandemic. The present study was designed to identify personality traits that predict such behavioral shifts in indoor versus outdoor leisure activities during the pandemic. The present study included 657 participants (*M*_age_ = 41.08) and measured 12 personality traits that a previous study reported were associated with health-protective behaviors during COVID-19. Our findings indicate that the rise in indoor leisure activities correlated with prosocial tendencies toward family and friends/acquaintances (but not strangers), self-centered interest, resilience, and Big Five personality traits. Conversely, the decline in outdoor activities was linked solely to prosociality toward family and friends/acquaintances. Further interaction analysis uncovered that prosocial tendencies toward close relations predicted increased indoor activities as an alternative to outdoor engagements. We concluded that prosociality promoted behavioral changes that significantly prevented infections in intimate others, and it could maintain personal well-being during the COVID-19 pandemic by facilitating behavior change.

## 1 Introduction

### 1.1 Behavioral changes in leisure activities and well-being

Leisure activities are pivotal in enhancing overall well-being (Newman et al., 2014; for a review, see Iso-Ahola and Baumeister, 2023). However, the onset of the COVID-19 pandemic has substantially curtailed these activities. Government-initiated travel restrictions and lockdowns aimed at containing the virus have curbed outdoor leisure activities, such as camping and shopping, potentially undermining well-being (van Bavel et al., 2020; Lee and Eom, 2023). Several studies highlight that reduced outdoor activities have had detrimental effects on well-being and mental health during this period (e.g., Lesser and Nienhuis, 2020; Jackson et al., 2021; Wright et al., 2021; Fernandez et al., 2022). Conversely, some research demonstrates that individuals have preserved their well-being during the pandemic (e.g., Pouso et al., 2020; Morse et al., 2021; Konishi et al., 2023). Specifically, Konishi et al. (2023) investigated leisure activity (related to health protective behaviors against COVID-19) shifts during the pandemic by comparing pre-pandemic (before 2019) and pandemic (in 2022) periods. Their findings correlate decreased outdoor leisure activities with a decline in well-being. Importantly, they found a positive association between deliberate increases in indoor leisure activities and enhanced well-being. Such findings are congruent with other studies indicating that individuals working from home or partaking in indoor creative activities have experienced greater well-being during the pandemic (Morse et al., 2021; Tuason et al., 2021). Konishi et al. (2023) study underscore the pivotal role of behavioral changes—specifically increasing indoor leisure activities as a substitute for outdoor leisure activities, on maintaining or improving well-being during the COVID-19 pandemic. Considering that well-being did not change during the pandemic, an increase of indoor leisure activities as a substitute for outdoor leisure activities observed in Konishi et al. (2023) could be interpreted as coping behaviors for maintaining well-being. Coping behaviors are important in situations where well-being is threatened (e.g., Skinner and Zimmer-Gembeck, 2007; Prayag et al., 2021). Therefore, it is worthy to examine what factors facilitate changes in leisure activities as coping behaviors.

### 1.2 Facilitators of behavioral changes

Society must understand the catalysts for behavioral changes crucial for well-being when anticipating potential future pandemics. Prior research underscores the significance of intentional leisure activity shifts for sustaining or elevating well-being as coping behaviors (Konishi et al., 2023). Still, the determinants that increase indoor leisure activities while decreasing outdoor activities remain elusive. In curbing infections, pinpointing facilitators of behavioral modifications that intentionally increase indoor leisure activities as a substitute for outdoor leisure activities becomes paramount. Given the intrinsic link between behavioral outcomes and personality (Ozer and Benet-Martínez, 2006), it is imperative to identify the personality traits promoting adaptive behaviors during the COVID-19 era. Hence, this study focuses on the relationship between personality traits and indoor and outdoor leisure activity shifts.

### 1.3 Role of personality traits during the COVID-19 pandemic

Preventing COVID-19 spread involves viewing increased indoor and reduced outdoor activities as health-protective behaviors analogous to mask-wearing, hand hygiene, and physical distancing. Recent research has investigated the interplay between personality traits and these protective behaviors during the COVID-19 pandemic (e.g., Jordan et al., 2021; Fang et al., 2022; Neumann-Böhme et al., 2022). These studies have identified various personality traits influencing health-protective behaviors, including prosociality, self-centeredness, resilience, the Big Five traits, infection aversion, self-control, locus of control dimensions, cultural values, curiosity, and optimism.

Notably, prosociality—actions intended to benefit others, such as following physical distance guidelines, staying home, purchasing face masks, and taking vaccination (Dovidio and Banfield, 2015)—have been linked to promoting health-protective behaviors (Campos-Mercade et al., 2021; Jordan et al., 2021; Schneider et al., 2021; Tse et al., 2022). Similarly, tendencies to engage in self-centered interests (i.e., pro-self) and antisocial personality (Blagov, 2021), including Machiavellianism, psychopathic tendencies, and maximization tendencies (Kokkoris, 2020) are associated with health-protective behaviors. Moreover, resilience, defined as the process of successfully adapting in the face of adversity, trauma, tragedy, threat, or significant threat sources (Palmiter et al., 2012), is associated with engagement in or adherence to health-protective behaviors during the pandemic (Fontes et al., 2022; Yıldırım and Arslan, 2022; see Zhang et al., 2022 for a review). Furthermore, other studies examined the association between the Big Five personality traits and health-protective behaviors during the COVID-19 pandemic. Results indicated that extraversion, conscientiousness, agreeableness, and openness subscale scores are positively associated with adherence to recommended protective measures. In contrast, Neuroticism is negatively related to these measures (Aschwanden et al., 2021; Kekäläinen et al., 2021; Kaspar and Nordmeyer, 2022; Moore et al., 2022; Otterbring and Festila, 2022). Additionally, previous studies have demonstrated the significant influence of the following personality traits, including aversion to infection (Stangier et al., 2021), self-control (Rodriguez et al., 2023), internal and external locus of control (Krampe et al., 2021), cultural values (Lu et al., 2021), curiosity (Losecaat Vermeer et al., 2022), and optimism (Sheetal et al., 2020) on health-protective behaviors.

Although studies have examined the relationship between changes in leisure activities and personality traits under the non-pandemic situation (e.g., Zimmer et al., 1997, Janke et al., 2006), to our best knowledge, no study has examined this relationship under the COVID-19 pandemic which required health-protective behaviors. Because Konishi et al. (2023) found the significant correlation between changes in leisure activities and health-protective behaviors under the COVID-19 pandemic, it is plausible that the personality traits related to health-protective behaviors can be influential predictors of changes in leisure activities. Therefore, the present study focused on these personality traits associated with health-protective behaviors in an exploratory manner.

### 1.4 Objective of the present study

The central aim of this study was to discern the personality attributes that foster increased indoor leisure pursuits as an alternative to outdoor engagements during the pandemic, which is crucial given the role of these behavioral shifts in improving well-being (Konishi et al., 2023). To address this, this study assessed 12 distinct personality scales from participants previously involved in Konishi et al. (2023), which assessed changes in indoor and outdoor leisure behaviors due to COVID-19. The personality scales we examined included prosociality, self-centered interest, resilience, the Big Five, aversion to infection, self-control, locus of control (both internal and external), cultural values, curiosity, and optimism, which have been associated with health-protective behaviors in previous studies. If increasing indoor leisure activities and reducing outdoor ones is a health-protective behavior, personality traits related to health-protective behaviors may also be correlated with behavioral changes in leisure activities. If certain personality traits correlate with an increased preference for indoor leisure and a reduced tendency for outdoor leisure, these traits can be critical factors in behavioral changes for maintaining or enhancing well-being. Moreover, we anticipate interactions between these specific traits and a reduction in outdoor leisure activities, predicting a rise in indoor leisure engagement.

## 2 Materials and methods

### 2.1 Participants

We conducted an Internet survey facilitated by a survey company MyVoice Communications, Inc. We surveyed 1,000 participants from a previous study by Konishi et al. (2023). The survey company sent an invitation email to these participants. This method ensured continuity by allowing us to utilize pre-existing data on behavioral changes in leisure activities. Of those invited via email, those who agreed to partake accessed a designated website to complete our survey. Participants were informed that the present survey is a follow-up study of the previously conducted survey. Participants also provided their informed consent to participate in this study again. We eliminated responses from participants who either consistently provided the same answer to all individual questions or completed the survey very quickly (less than 150°s). After these exclusions, we collected valid data from 657 participants (310 men and 347 women; average age = 41.08°years, *SD* = 10.62; age ranging from 21 to 59°years). Because the data was collected and anonymized (with lossy coding) by a survey company, we did not receive any personally identifiable information. A four-month gap separated our survey from the one conducted in Konishi et al. (2023). The data and analysis codes can be accessed through the Open Science Framework^1^^,^^2^.

### 2.2 Behavioral changes in leisure activities during COVID-19

Konishi et al. (2023) previously reported data detailing shifts in leisure activities during the pandemic. Participants in that study assessed changes in their indoor and outdoor leisure pursuits in 2022 (during the pandemic) relative to their 2019 habits (pre-pandemic) using a 5-point Likert scale (1 representing a significant decrease, and 5 indicating a substantial increase). The behavioral shifts in 11 specified indoor (like home workouts and streaming services usage) and 11 outdoor activities (such as visits to bars/clubs or traveling) were notably associated with the COVID-19 Coping Behavior Scale (Wakashima et al., 2020). This scale evaluates care, stockpiling, and health monitoring habits in everyday life. Therefore, these 22 activities served as proxies for changes induced by the pandemic. Subsequently, we averaged and converted the ratings from the 5-point scale to a scale spanning from −2 (markedly decreased) to 2 (significantly increased). The compiled data revealed an increase in indoor activities (*M* = 0.23, *SD* = 0.59) and a downturn in outdoor pursuits (*M* = −0.70, *SD* = 0.70). These changes positively correlated with measures of well-being. In the current study, the indices of indoor and outdoor activities of the 657 participants (ranging from −2 to 2) were analyzed using correlation and multiple regression analyses to identify their association with personality traits.

### 2.3 Procedure and materials

After providing informed consent, participants in the current survey responded to 12 established scales assessing prosociality, self-centered interest, resilience, the Big Five, aversion to infection, self-control, internal and external locus of control, cultural values, curiosity, and optimism.

#### 2.3.1 Prosociality

We assessed prosociality using the Self-Report Altruism Scale Distinguished by the Recipient (SRAS-DR; Oda et al., 2013). The SRAS-DR assesses the frequency of seven types of altruistic behaviors (i.e., family and friends/acquaintances, and strangers). The scale asked participants to rate the frequency of altruistic behaviors. Responses are made using a 5-point Likert scale ranging from 1 (*never*) to 5 (*very often*). Examples of items are “I have taken care of a family member when he or she was sick” (for family), “I have accompanied a friend to a place where he or she wanted to go” (for friends/acquaintances), and “I have helped strangers stand up when they fell over on the street” (for strangers). In the present study, the Cronbach’s alpha for the entire scale was 0.91, and the Cronbach’s alphas for the three subscales were 0.84 (altruism toward family), 0.82 (altruism toward friends/acquaintances), and 0.88 (altruism toward strangers).

#### 2.3.2 Self-centered interest

This dimension, the tendency to put one’s interests first, was assessed using the Dark Triad Dirty Dozen (DTDD; Jonason and Webster, 2010; Tamura et al., 2015 for the Japanese version). Participants indicated the extent to which they agreed with nine items assessing narcissism (e.g., “I tend to want others to admire me”) and nine items assessing Machiavellianism (e.g., “I tend to manipulate others to get my way”). Responses were made using a 4-point Likert scale ranging from 1 (*not at all agree*) to 4 (*very much agree*). The items were averaged to create an index of narcissism (Cronbach’s alpha = 0.82) and Machiavellianism (alpha = 0.77). The DTDD also includes psychopathy characteristics, which we did not assess to reduce the survey participants’ burden. We also evaluated self-centered interest using the Maximization Inventory (MI; Turner et al., 2012; Ishiguro, 2021 for the Japanese version) by using the subscale of 12 alternative search items (e.g., “If a store doesn’t have exactly what I’m shopping for, then I will go somewhere else”). Responses were made using a 5-point Likert scale ranging from 1 (*not at all*) to 5 (*quite a lot*). We averaged the items to develop the index of Maximization (Cronbach’s alpha = 0.88).

#### 2.3.3 Resilience

We used two scales to assess these tendencies. The Bidimensional Resilience Scale developed in Japan (BRS; Hirano, 2010) consists of 12 items measuring innate resilience (e.g., “I think that things will work out on most occasions in any case”) and nine items measuring acquired resilience (e.g., “I am good at understanding others’ ways of thinking”). Responses were made using a 5-point Likert scale ranging from 1 (*strongly disagree*) to 5 (*strongly agree*). Cronbach’s alphas of innate resilience and acquired resilience were 0.88 and 0.81, respectively. We also measured resilience using the Adolescent Resilience Scale (ARS; Oshio et al., 2003). Adolescent resilience is defined as a psychological trait promoting recovery from mental depression. This scale consists of seven novelty-seeking items (e.g., “I seek new challenges.”; Cronbach’s alpha = 0.73), nine emotion regulation items (e.g., “I think I can control my emotions”; alpha = 0.79), and five positive future orientation items (e.g., “I think I have a positive bright future;” alpha = 0.86). Responses were made using a 5-point Likert scale ranging from 1 (*definitely no*) to 5 (definitely yes).

#### 2.3.4 Big five

We assessed the Big Five personality traits using the Japanese version of the Ten Item Personality Inventory (TIPI-J; Oshio et al., 2012). The scale consists of two items for each trait: extraversion (correlation of two items; *r* = 0.39), agreeableness (*r* = 0.28), conscientiousness (*r* = 0.44), openness (*r* = 0.32), and neuroticism (*r* = 0.39). Participants were instructed to rate these items on a 7-point Likert scale ranging from 1 (*completely disagree*) to 7 (*strongly agree*). This scale is frequently used for its relative brevity despite not being designed for high internal consistency.

#### 2.3.5 Aversion to infection

We measured perceived vulnerability to disease using the Japanese version of the Perceived Vulnerability to Disease scale (PVD-J; Fukukawa et al., 2014), developed based on Duncan et al. (2009). The PVD-J consists of 15 items and two subscales: perceived infectability (e.g., “I am more likely than the people around me to catch an infectious disease”) and germ aversion (e.g., “I prefer to wash my hands pretty soon after shaking someone’s hand”). Responses were made on a 7-point Likert scale ranging from 1 (*strongly disagree*) to 7 (*strongly agree*). The Cronbach’s alpha coefficients for perceived infectability and germ aversion were 0.79 and 0.73, respectively.

#### 2.3.6 Self-control

We accessed self-control using the Japanese version (Ozaki et al., 2016) of the Brief Self-Control Scale (BSCS; Tangney et al., 2004). The scale consists of 13 items (e.g., “People would say that I have very strong self-discipline”). Responses were made using a 5-point Likert scale ranging from 1 (completely disagree) to 5 (*strongly agree*). The Cronbach’s alpha for the present study was 0.80.

#### 2.3.7 Internal and external locus of control

These were measured using the Locus of Control scale (LoC-J; Kanbara et al., 1982), which evaluates both internal and external control orientations. Participants rated nine items corresponding to internal control (e.g., “Do you think you can become a good person if you work hard”; Cronbach’s alpha = 0.80) and nine items corresponding to external control (e.g., “Do you believe that your life is determined by fate;” alpha = 0.72). Responses were made using a 4-point Likert scale ranging from 1 (strongly disagree) to 7 (*strongly agree*).

#### 2.3.8 Cultural values

To determine cultural self-construal, we employed Independent and Interdependent Self-construal assessed by a Japanese scale (Takata, 1999). Participants were asked to rate their level of agreement with ten independent items (e.g., “I make decisions about things on my own;” Cronbach’s alpha = 0.83) and ten interdependent items (e.g., “I am concerned about the way others look at me;” alpha = 0.78). Responses were made on a 7-point Likert scale ranging from 1 (*completely disagree*) to 7 (*strongly agree*).

#### 2.3.9 Curiosity

We assessed curiosity using the Epistemic Curiosity Scale (ECS; Nishikawa and Amemiya, 2015). This scale consists of two subscales: 6 items related to diverse curiosity (e.g., “I am very interested in events that no one has done before”) and six items on specific curiosity (e.g., “When I learn something, I like to research it thoroughly”). Responses were made on a 5-point Likert scale ranging from 1 (*strongly disagree*) to 5 (*strongly agree*). The Cronbach’s alpha coefficients for diverse curiosity and specific curiosity were 0.90 and 0.85, respectively.

#### 2.3.10 Optimism

We assessed optimism using the Japanese version of the Optimism and Pessimism Scale (OPS; Toyama, 2013), first developed by Scheier and Carver (1985). The scale consists of 10 items assessing optimism (e.g., “I think my future is blessed”; Cronbach’s alpha = 0.93) and ten items measuring pessimism (e.g., “I think my future is bleak”; alpha = 0.92). Responses are made on a 4-point Likert scale ranging from 1 (*completely disagree*) to 4 (*strongly agree*).

### 2.4 Data analysis

We computed Pearson’s correlation coefficients to determine the relationships between various personality traits and indoor/outdoor activity indices. Multiple regression analyses were undertaken after identifying personality traits that exhibited significant correlations with indoor and outdoor activity indices. In these analyses, an interaction term combining the personality trait with the outdoor activity index served as the independent variable. In contrast, the indoor activity index was entered as the dependent variable. For these statistical procedures, we utilized the psych package (Revelle, 2020), the interactions package (Long, 2019), and the jtools package (Long, 2022) within the R statistical language (Version 3.6.3; R Core Team, 2020).

## 3 Results

### 3.1 Association between personality traits and indoor/outdoor activity indices

We analyzed correlations between personality traits and shifts in indoor/outdoor leisure activities during the COVID-19 pandemic, as assessed by Konishi et al. (2023). As shown in Table 1, changes in indoor leisure behaviors were significantly and positively associated with multiple personality traits, suggesting that these personality traits predict an increase in indoor leisure activities compared to 2019 (before the pandemic). In contrast, changes in outdoor leisure activities were significantly and negatively related to only two personality traits, suggesting that two personality traits predict a decrease in outdoor leisure activities compared to 2019 (see Materials and methods section “2.2 Behavioral changes in leisure activities during COVID-19” for more information on the leisure activity indexes and details).

**TABLE 1:** Correlation between personality traits and changes in leisure activities.

	Indoor activities			Outdoor activities		
	***r***	***p***	**95% CI**	***r***	***p***	**95% CI**
**Prosociality**						
Altruism toward family	**0.203**	<0.001	[0.13, 0.28]	**−0.175**	<0.001	[−0.25, −0.10]
Altruism toward friends/acquaintances	**0.213**	<0.001	[0.14, 0.29]	**−0.144**	0.006	[−0.22, −0.07]
Altruism toward strangers	−0.026	1.00	[−0.1, 0.05]	−0.093	0.384	[−0.17, −0.02]
**Self-centered interest**						
Maximization	**0.175**	<0.001	[0.1, 0.25]	0.025	1.00	[−0.05, 0.10]
Machiavellianism	**0.131**	0.014	[0.06, 0.21]	0.041	1.00	[−0.04, 0.12]
Narcissism	−0.073	0.732	[−0.15, 0.00]	0.098	0.278	[0.02, 0.17]
**Resilience**						
Innate resilience	**0.162**	0.001	[0.09, 0.24]	−0.052	1.00	[−0.13, 0.03]
Acquired resilience	**0.230**	<0.001	[0.16, 0.30]	−0.105	0.181	[−0.18, −0.03]
Novelty seeking	**0.190**	<0.001	[0.12, 0.26]	0.000	1.00	[−0.08, 0.08]
Emotional regulation	0.104	0.116	[0.03, 0.18]	−0.069	1.00	[−0.15, 0.01]
Positive future orientation	**0.126**	0.021	[0.05, 0.20]	−0.072	1.00	[−0.15, 0.01]
**Big five**						
Extraversion	0.005	1.00	[−0.07, 0.08]	−0.070	1.00	[−0.15, 0.01]
Agreeableness	**0.212**	<0.001	[0.14, 0.28]	−0.069	1.00	[−0.15, 0.01]
Conscientiousness	0.101	0.137	[0.02, 0.18]	−0.032	1.00	[−0.11, 0.05]
Openness	0.006	1.00	[−0.07, 0.08]	0.063	1.00	[−0.01, 0.14]
Neuroticism	0.027	1.00	[−0.05, 0.10]	0.037	1.00	[−0.04, 0.11]
**Aversion to infection**						
Perceived infectability	−0.006	1.00	[−0.08, 0.07]	−0.007	1.00	[−0.08, 0.07]
Germ aversion	0.014	1.00	[−0.06, 0.09]	−0.002	1.00	[−0.08, 0.08]
**Locus of control**						
Internal control	−0.008	1.00	[−0.09, 0.07]	0.034	1.00	[−0.04, 0.11]
External control	0.112	0.066	[0.04, 0.19]	−0.068	1.00	[−0.14, 0.01]
**Cultural values**						
Interdependent	0.027	1.00	[−0.05, 0.10]	0.104	0.190	[0.03, 0.18]
Independent	−0.064	1.00	[−0.14, 0.01]	0.058	1.00	[−0.02, 0.14]
**Curiosity**						
Diverse curiosity	**0.144**	0.004	[0.07, 0.22]	0.017	1.00	[−0.06, 0.09]
Specific curiosity	**0.173**	<0.001	[0.10, 0.25]	−0.017	1.00	[−0.09, 0.06]
**Optimism**						
Optimism	0.059	1.00	[−0.02, 0.14]	−0.075	1.00	[−0.15, 0.00]
Pessimism	−0.048	1.00	[−0.12, 0.03]	0.029	1.00	[−0.05, 0.11]
Self-control	0.081	0.503	[0.00, 0.16]	−0.053	1.00	[−0.13, 0.02]

Bolded values are statistically significant at *p* < 0.05. Probability values were adjusted for multiple tests using the Holm correction. R stands for correlation coefficient, and CI denotes confidence intervals. “Indoor activity” refers to variations in leisure activities at home, whereas “outdoor activity” pertains to changes in activities conducted outside the home.

The indoor activity index displayed positive correlations with the following traits: altruism toward family [*r*(654) = 0.20, *p*°<°0.001], altruism toward friends/acquaintances [*r*(654) = 0.21, *p*°<°0.001], maximization [*r*(654) = 0.18, *p*°<°0.001], Machiavellianism [*r*(654) = 0.13, *p* = 0.014], both innate [*r*(654) = 0.16, *p* = 0.001] and acquired resilience [*r*(654) = 0.23, *p*°<°0.001] novelty seeking [*r*(654) = 0.19, *p*°<°0.001], positive future orientation [*r*(654) = 0.13, *p* = 0.021], agreeableness [*r*(654) = 0.21, *p* < 0.001], and both diverse [*r*(654) = 0.14, *p* = 0.01], and specific curiosity [*r*(654) = 0.17, *p* < 0.001]. These results suggest that individuals exhibiting heightened prosociality toward their family and friends/acquaintances, self-centered interest, resilience, agreeableness, and curiosity tended to intensify their indoor leisure engagements during the pandemic.

The outdoor activity index was significantly associated only with prosociality, altruism toward family [*r*(650) = −0.18, *p* < 0.001], and altruism toward friends/acquaintances [*r*(650) = −0.14, *p* = 0.006]. This finding implies that individuals with robust prosocial inclinations toward their family and friends/acquaintances were more likely to curtail their outdoor leisure activities during the pandemic.

### 3.2 Prosociality and behavioral adjustments

The correlational analyses between personality traits and behavioral changes in leisure activities revealed that prosociality, whether directed toward family and friends/acquaintances, was the sole trait significantly related to an increase in indoor leisure activities and a decrease in outdoor leisure activities. Consequently, we further explored whether prosocial tendencies could predict an increase in indoor activities as an alternative to outdoor activities during the pandemic. We conducted multiple regression analyses, including the interaction between each subscale of prosociality (i.e., toward family and friends/acquaintances) with the outdoor activity index and the indoor activity index as the dependent variables after controlling for gender, age, marital status, and income (see Table 2). Our findings indicate that interactions both toward family and friends/acquaintances and the outdoor activity index significantly predicted the indoor activity index [altruism toward family; β = −0.07, *t*(650) = −3.25, *p* = 0.001, altruism toward friends/acquaintances; β = −0.05, *t*(650) = −2.40, *p* = 0.017].

**TABLE 2:** Results of multiple regression analysis: Interactions between prosociality and outdoor activities influencing indoor activities.

	β	*t*	95% CI
**Altruism toward family**			
(Intercept)	0.18***	4.42	[0.15, 0.21]
Gender (0; male, 1; female)	−0.01	−0.12	[−0.04, 0.02]
Age	−0.01	−0.22	[−0.02, 0.01]
Income	0.01	0.58	[0, 0.03]
Marriage (0; unmarried. 1; married)	0.12*	2.42	[0.09, 0.15]
Outdoor activity	−0.02	−0.91	[−0.04, −0.01]
Altruism toward family	0.13***	5.68	[0.11, 0.14]
Outdoor activity × altruism toward family	−0.07**	−3.25	[−0.08, −0.05]
**Altruism toward friends/acquaintances**			
(Intercept)	0.18***	4.46	[0.16, 0.21]
Gender (0; male, 1; female)	0.04	0.89	[0.01, 0.07]
Age	0.00	−0.01	[−0.02, 0.02]
Income	0.01	0.61	[0, 0.03]
Marriage (0; unmarried. 1; married)	0.08	1.68	[0.05, 0.12]
Outdoor activity	−0.02	−0.84	[−0.03, 0]
Altruism toward friends/acquaintances	0.13***	5.65	[0.11, 0.14]
Outdoor activity × altruism toward friends/acquaintances	−0.05*	−2.40	[−0.06, −0.03]

CI stands for confidence intervals. “Indoor activity” refers to variations in leisure activities conducted at home, whereas “outdoor activity” pertains to fluctuations in activities outside the home. Significance levels are as follows:

*** for *p* < 0.001, ** for *p* < 0.01, and * for *p* < 0.05.

**FIGURE 1:**
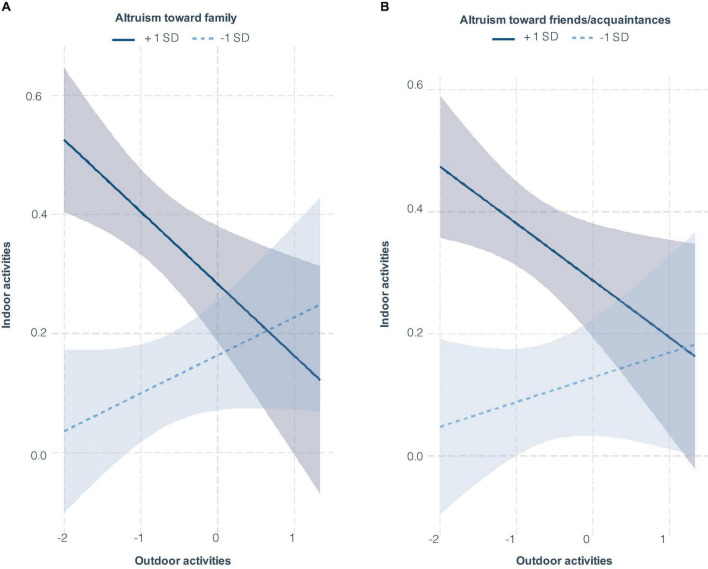
Impact of altruism on activity preferences. **(A)** depicts the relationship between altruism toward family and the balance of indoor versus outdoor activities. **(B)** shows the correlation for altruism toward friends/acquaintances. The vertical axes in both panels illustrate the variation in indoor activities, with values greater than zero indicating an increase and values less than zero indicating a decrease. Similarly, the horizontal axes represent variations in outdoor activities. The dark blue solid line represents individuals with strong altruism (greater than 1 SD above the mean), while the light blue dashed line indicates individuals with weak altruism (less than 1 SD below the mean). Shaded regions denote 95% confidence intervals.

Figure 1 illustrates the simple slope analysis results revealed a negative relationship between outdoor and indoor activity indices when prosociality increased (Altruism toward family; β = −0.12, *t* = −2.86, *p* < 0.001 shown as a solid line in Figure 1A, altruism toward friends/acquaintances; β = −0.09, *t* = −2.29, *p* = 0.02 shown as a solid line in Figure 1B.). In contrast, a weaker prosocial inclination did not result in a significant correlation between these indices (altruism toward family; β = 0.06, *t* = 1.51, *p* = 0.13 shown as a dashed line in Figure 1A, altruism toward friends/acquaintances; β = 0.04, *t* = 0.94, *p* = 0.35 shown as a dashed line in Figure 1B).

## 4 Discussion

Research by Konishi et al. (2023) revealed that a decrease in outdoor leisure activities coupled with an increase in indoor ones plays a pivotal role in maintaining or enhancing well-being during the COVID-19 pandemic. The current study further explored the correlation between behavioral shifts in outdoor/indoor leisure activities and personality traits. Our findings indicate that an increase in indoor leisure activities correlates significantly with prosociality (toward family or friends/acquaintances), self-centered interest, resilience, agreeableness, and curiosity. Conversely, the decline in outdoor activities is significantly associated only with prosociality toward family or friends/acquaintances. Notably, we identified a meaningful interaction effect between changes in outdoor activities and prosociality affecting indoor activities, suggesting the role of prosociality toward family or friends/acquaintances in substituting indoor for outdoor leisure activities during the pandemic.

### 4.1 Prosociality and behavioral changes

Among the diverse personality traits we measured, only prosociality toward family or friends/acquaintances was connected to both increased indoor and decreased outdoor leisure activities. This suggests that individuals with higher altruism toward family and/or friends/acquaintances likely reduced outdoor activities while increasing indoor activities. Though the association was not significant, those with weaker altruism showed the opposite trend. Our findings propose that prosocial tendencies do not merely increase indoor activities but promote indoor activities as a substitute for outdoor leisure activities. This might represent an adaptive lifestyle shift to prevent COVID-19 transmission and foster well-being. Past research underscores the rise of prosocial behaviors during societal crises (e.g., Rodríguez et al., 2006; Zaki, 2020; Hellmann et al., 2021; Haller et al., 2022). Furthermore, prosociality encourages health-protective behaviors that directly cope with COVID-19 (Fang et al., 2022; Neumann-Böhme et al., 2022). This study presents a novel perspective by demonstrating that prosociality encourages health-protective behaviors (e.g., a decrease in outdoor leisure activities) and leads to adaptive changes in leisure activities (i.e., an increase in indoor leisure activities). Engaging in indoor leisure activities while seemingly achieving only personal goals like increasing own well-being might also be accompanied by caring for others and preventing the spread of COVID-19, especially in individuals whose prosociality is high. Restricting outdoor leisure activities helps prevent the spread of COVID-19, even though it can also reduce well-being. Prosociality, instead, has a facilitatory influence on engaging in alternative behaviors (i.e., indoor leisure activities) to maintain and improve overall well-being.

### 4.2 Prosociality: Family and friends/acquaintances vs. strangers

Interestingly, while prosociality toward family or friends/acquaintances fostered adaptive changes, such tendencies toward strangers did not. Why does prosociality toward immediate circles, but not strangers, influence leisure behaviors? The answer may lie in the balance of costs and benefits. Diminishing outdoor activities can sacrifice personal well-being. The rewards stemming from these sacrifices likely benefit family and friends/acquaintances more than strangers. Consequently, individuals with strong prosocial leanings toward family and friends/acquaintances might be more attuned to the protective benefits of decreasing outdoor leisure activities, such as preventing infection of family or friends/acquaintances. However, even if prosociality toward strangers is potent, the perceived societal benefits of their sacrifice (i.e., reducing the spread of infection throughout society) might not outweigh the personal costs of decreasing outdoor leisure activities. This finding aligns with literature suggesting that prosocial actions typically render higher costs yet greater rewards when directed at close relations compared to distant ones (Rachlin and Jones, 2008, see Kurzban et al., 2015 for a review). The present study provides new evidence that altruism toward family and friends/acquaintances may be crucial for health-protective behaviors. Previous studies did not elucidate this finding because they did not separately examine recipients of prosociality.

However, previous studies have reported associations between prosociality toward strangers (e.g., altruistic behaviors in economic games or donations) and health-protective behaviors (e.g., Neumann-Böhme et al., 2022). This inconsistency might arise from the differences in the target behaviors examined in the present study (i.e., decrease in outdoor leisure activities) compared to those in previous research (i.e., health-protective behaviors such as wearing masks). Decreasing outdoor activities could impose higher costs, such as reducing well-being, compared to other health-protective behaviors like mask-wearing. Previous research has indicated that higher costs can diminish the influence of prosociality in promoting health-protective behaviors. For instance, while fostering an understanding of the social benefits of vaccination may boost the intent to vaccinate, this effect diminishes when the perceived cost of vaccination is high (Böhm and Betsch, 2022). Consequently, the absence of a significant association between prosociality toward strangers and behavioral changes in the current study might be attributed to the higher costs associated with reducing outdoor leisure activities.

### 4.3 Prosociality and pandemic interventions

The present study’s results could offer valuable insights for interventions in potential future pandemics. Prior research has demonstrated that messages promoting health-protective behaviors are most effective when they are prosocial and directed at individuals (Jordan et al., 2021). However, these studies have typically examined prosocial messages in a general sense (i.e., advising to avoid contracting and spreading COVID-19) rather than messages specifically aimed at family and friends. Based on our findings, messages targeting family and friends might promote health-protective behaviors more effectively. Moreover, messages focused on this group might encourage health-protective actions and facilitate adaptive behavioral changes to boost well-being. In light of these findings, interventions during future pandemics, including those initiated by governments, might benefit from emphasizing prosociality toward family and friends in their messaging. Further, such intervention may be effective to increase health-protective behaviors even in non-pandemic situation. This issue may be worthy to explore in future studies.

### 4.4 Other personality traits and behavioral changes

The current study found a correlation between an increase in indoor leisure activities with prosociality and various personality traits, including self-centered interest, resilience, Big Five factors, and curiosity. Although indoor activities might not directly ward off infections, they could contribute to sustaining or enhancing well-being. Notably, individuals with strong tendencies toward maximization and Machiavellian traits linked to self-centered interests (Christie and Geis, 1970; Schwartz et al., 2002) seemed to bolster indoor activities, potentially maximizing benefits amidst COVID-19 behavioral restrictions. Resilience, another trait associated with an increase in indoor activities, suggests these activities might serve as coping mechanisms (Masten et al., 1990) against pandemic-induced stress. Agreeableness, a component of the Big Five, was also tied to increased indoor pursuits. This aligns with earlier research indicating that agreeableness correlates with adherence to social distancing (Moore et al., 2022) and health-protective behaviors (Otterbring and Festila, 2022). Conversely, curiosity is known to mediate well-being positively and coping with social constraints in the context of COVID-19 by reducing loneliness (Losecaat Vermeer et al., 2022). The present result may similarly indicate that increased indoor leisure activities can decrease loneliness. It is imperative to underscore that these traits were exclusively linked to increased indoor activities, not decreased outdoor ones. This finding suggests that the rise in indoor activities is not merely a substitute for the decrease in outdoor leisure activities. Instead, during the COVID-19 outbreak, the likelihood of encountering new indoor pastimes might have increased, and specific personality traits might incline individuals toward embracing such novel experiences, regardless of infection prevention.

### 4.5 Limitations

Several limitations should be considered when interpreting the results of this study. Firstly, this study explored associations between behavioral shifts and personality traits without establishing a direct causal link. Personality was gauged post-pandemic, making it challenging to dismiss potential changes in these traits due to COVID-19 experiences. As such, our data does not offer a clear causal relationship between behavioral shifts and personality traits. Secondly, our study was confined to Japan, where the pandemic-driven behavioral restrictions were relatively lenient compared to nations under stringent lockdowns. It should be noted that in other countries (such as the U.S., EU, and China), the lockdown was a very solemn restriction on going out, whereas in Japan, the restriction on going out was only recommended, and therefore participants had more options for outdoor leisure activities. Consequently, changes in leisure activities in Japan may be more heterogeneous than in other countries, influencing their relationship with personality traits.

## 5 Conclusion

Previous work indicated that consciously increasing indoor activities as an alternative to outdoor ones positively influenced well-being during the COVID-19 crisis (Konishi et al., 2023). Extending these findings, this research suggests that prosociality—especially toward family, friends, and acquaintances—can predict the intentional increase of indoor leisure activities as a substitution for outdoor leisure activities. This underscores the dual role of prosociality during pandemics: safeguarding others through infection prevention and preserving individual well-being through adaptive behaviors.

In previous studies, the behavior of increasing indoor activities as a substitute for outdoor activities was interpreted as coping behaviors. The present study provided further understanding of coping behaviors under the pandemic. That is, although coping behaviors are considered to be related to one’s benefits, changes in leisure activities during the pandemic are also motivated by benefits for others. Therefore, toward understanding of behavioral changes in pandemic situations, focuses not only on individual motivations but also on social motivations (especially to family and friends) are important. This finding can be beneficial to public policy productions against infections and disasters, for example, by providing messages that encourage behavioral changes not only in terms of targeting individuals, but also in terms of targeting their family and friends.

## Data availability statement

The datasets presented in this study can be found in online repositories. The names of the repository/repositories and accession number(s) can be found below: Open Science Framework (https://osf.io/7amdu/).

## Ethics statement

Ethical approval was not required for the studies involving humans because we did not record any personal data of participants in accordance with the local legislation and institutional requirements. The studies were conducted in accordance with the local legislation and institutional requirements. The participants provided their written informed consent to participate in this study.

## Author contributions

NK: Conceptualization, Data curation, Formal Analysis, Writing – original draft, Writing – review & editing. MK: Conceptualization, Writing – review & editing, Writing – original draft. YT: Conceptualization, Writing – review & editing, Writing – original draft.

## Footnotes

1 The present study was not preregistered and was conducted in an exploratory approach to investigate what personality traits can be associated.

2
https://osf.io/7amdu/
